# Automatic activation of alcohol cues by child maltreatment related words: a replication attempt in a different treatment setting

**DOI:** 10.1186/s13104-016-2324-8

**Published:** 2017-01-03

**Authors:** Nadine Potthast, Frank Neuner, Claudia Catani

**Affiliations:** Department of Psychology, Bielefeld University, Postbox 100 131, 33501 Bielefeld, Germany

**Keywords:** Child abuse, Emotional maltreatment, Alcohol dependence, Underlying mechanisms, Priming, Associative memory network, Replication, Qualified detoxification sample

## Abstract

**Background:**

A growing body of research attempts to clarify the underlying mechanisms of the association between emotional maltreatment and alcohol dependence (AD). In a preceding study, we found considerable support for a specific priming effect in subjects with AD and emotional abuse experiences receiving alcohol rehabilitation treatment. We concluded that maltreatment related cues can automatically activate an associative memory network comprising cues eliciting craving as well as alcohol-related responses. Generalizability of the results to other treatment settings remains unclear because of considerable differences in German treatment settings as well as insufficiently clarified influences of selection effects. As replication studies in other settings are necessary, the current study aimed to replicate the specific priming effect in a qualified detoxification sample.

**Results:**

22 AD subjects (n = 10 with emotional abuse vs. n = 12 without emotional abuse) participated in a priming experiment. Comparison data from 34 healthy control subjects were derived from the prior study. Contrary to our hypothesis, we did not find a specific priming effect.

**Conclusions:**

We could not replicate the result of an automatic network activation by maltreatment related words in a sample of subjects with AD and emotional abuse experiences receiving qualified detoxification treatment. This discrepancy might be attributed to reasons related to treatment settings as well as to methodological limitations. Future work is required to determine the generalizability of the specific priming effect before valid conclusions regarding automatic activation can be drawn.

## Background

Recent research indicates that there is a link between child maltreatment and alcohol dependence (AD; [1, 2]) and predominantly between emotional types of maltreatment and AD [3–5]. However, the underlying mechanisms still need to be clarified. Findings of laboratory-based experimental investigations suggest that trauma-related cues are able to increase craving, presumably by eliciting negative emotions [6, 7]. Based on memory network models [8], it can be assumed that the processes underlying the induction of craving by trauma-related cues are very fast, automatic and largely unconscious. Trauma relevant cues might automatically activate the memory network thus stimulating craving [9]. Accordingly, Sherman [10] found that trauma exposed smokers showed a significant priming effect within a modified Stroop task, when trauma related pictures preceded smoking related words. There is reason to assume that maltreatment related cues automatically activate an associative memory network comprising cues eliciting craving as well as alcohol-related responses. To our knowledge, there is only one study examining this assumption so far [11], which was conducted by our group. Using a priming paradigm, we examined whether the associative memory network in subjects with AD and experiences of childhood emotional abuse could be activated by child maltreatment related cues. The AD sample consisted of 49 individuals who were receiving alcohol rehabilitation treatment in Germany. As expected, we found considerable support for a specific priming effect in the emotionally abused alcoholics. We concluded that cues related to child maltreatment can automatically activate the associative memory network in AD subjects with emotional abuse experiences.

Considering the selective sample of patients enrolled in long-term rehabilitation treatment along with the overall complexity of the German addiction treatment system, generalizability of the results remains unclear. The German addiction treatment system comprises several kinds of treatment settings with somatic detoxification treatment, qualified detoxification treatment and rehabilitation treatment being the most frequent. While somatic detoxification treatment focuses on physical withdrawal only, qualified detoxification treatment is predominantly considered to motivate patients to engage in a subsequent long-term rehabilitation treatment [12]. However, only a limited number of patients actually proceed to rehabilitative treatment with transition rates ranging from 4 to 21% [13–16], suggesting considerable selection effects. Yet, studies are lacking which provide information about the extent and characteristics of potential selection effects by comparing patients participating in rehabilitation programs to those who decide against it. To our knowledge, there is only one report, which peripherally addressed this issue. Results indicated that patients proceeding to rehabilitation had a more obsessive consumption pattern, more negative consequences of drinking and a more severe psychopathological burden as compared to subjects quitting after detoxification [17]. Altogether, differences in treatment settings as well as influences of selection effects require clarification.

Against this background, it remains unclear, if the finding of the automatic activation of the associative memory network by child maltreatment related cues can be generalized to treatment settings other than rehabilitation treatment. The present study aimed to replicate the finding in patients undergoing qualified detoxification therapy after having completed physical withdrawal. We presumed that the automatic network activation occurs independently of treatment setting. Hence, we proposed to find the priming effect of maltreatment related words on alcohol words in a qualified detoxification sample, as well. Analogous to our prior work, we used socially threatening cues as well as physically threatening cues to differentiate between emotional and physical maltreatment. As we assume that stimuli associated with physical abuse are integrated in the same associative network as stimuli associated with emotional abuse, we expected to find the priming effect in subjects with emotional abuse experiences for words related to emotional abuse as well as for words related to physical abuse.

## Methods

### Participants

The AD sample consisted of 27 subjects receiving short-term treatment in a German day-unit immediately after finishing physical detoxification. For inclusion in the study, AD participants in both samples had to be aged over 18 and had to meet DSM-IV criteria for AD [18] as principal diagnosis. Exclusion criteria were current comorbid substance use disorder, current or lifetime psychosis, and severe cognitive problems. Patients meeting inclusion criteria were asked to participate in the study at the beginning of their treatment. Interested patients received a complete description of the study and were invited to the clinical interview. All participants of the interview, who were native German speakers without neurological problems or dyslexia, were asked to participate additionally in the experimental study. Interested subjects received a description of the experimental design and an appointment was scheduled within seven days after the interview. Overall, five AD participants had to be excluded. Three participants did not reach the cut-off score in the AUDIT and two participants turned out to have cannabis dependence as principal diagnosis. The whole sample (n = 22) was divided into two subsamples according to the presence of emotional abuse (AD + EA vs. AD + no EA) based on the subscale *emotional abuse* of the *Childhood Trauma Questionnaire* (CTQ; [19]; see below).

Comparison data from 34 healthy control subjects were derived from a previously published study [11]. Table 1 provides an overview of the socio-demographic and clinical characteristics of the AD participants with emotional abuse (n = 10), AD patients without emotional abuse (n = 12), and the control participants (n = 34) as well as significant group differences. The control group was significantly younger. However, there were no differences between the two patients groups.

**Table 1:** Subjects’ sociodemographic and clinical characteristics

	AD + EA (n = 10)	AD + no EA (n = 12)	no AD (n = 34)
Age, M (SD)	43.60 (5.60)^a^	44.33 (13.11)^a^	34.24 (13.34)^b^
Gender, % male (n)	70.0 (7)^a^	58.3 (7)^a^	35.3 (12)^a^
Family status, % single (n)	50.0 (5)^a^	58.3 (7)^a^	67.6 (23)^a^
Education, % graduation and higher (n)	100.0 (10)^a^	100.0 (12)^a^	100.0 (34)^a^
Employment, % unemployed (n)	30.0 (3)^ab^	58.3 (7)^a^	8.8 (3)^b^
Medication, % psychopharmacological treatment (n)	60.0 (6)^a^	75.0 (9)^a^	5.9 (2)^b^
Medication for alcoholism, % taking disulfiram, naltrexone or acamprosate (n)	30.0 (3)^a^	8.3 (1)^ab^	0.0 (0)^b^
Psychotherapeutic treatment, % lifetime (n)	50.0 (5)^a^	58.3 (7)^a^	35.3 (12)^a^
*Childhood trauma questionnaire*			
Emotional abuse, % above threshold (n)	100.0 (10)^a^	0.0 (0)^b^	17.06 (6)^b^
Emotional neglect, % above threshold (n)	60.0 (6)^a^	8.3 (1)^b^	23.5 (8)^ab^
Physical abuse, % above threshold (n)	70.0 (7)^a^	16.7 (2)^b^	14.7 (5)^b^
Sexual abuse, % above threshold (n)	10.0 (1)^a^	8.3 (1)^a^	14.7 (5)^a^
Alcohol use disorders identification test, M (SD)	22.30 (5.01)^a^	22.25 (7.06)^a^	3.03 (2.07)^b^
Comorbid axis I psychiatric disorder, % yes (n)	90.9 (9)^a^	75.0 (9)^a^	20.7 (6)^b^

*AD* *+* *EA* subjects with alcohol dependence and emotional abuse experiences; *AD* *+* *no EA* subjects with alcohol dependence without emotional abuse experiences; *no AD* control subjects; indices represent the results of pair-wise group comparisons using the t-tests for continuous variables and the Chi-Quadrat test for dichotomous variables

Different indices indicate significant differences on *p* < 0.05

Power analysis suggests that a total sample size of N = 48 is needed to replicate the priming effect with an expected medium effect size of f = 0.30 assuming a power of ≥0.80 and an alpha level of 0.05 (G*Power, Version 3.1.9.2, University Düsseldorf, Germany).

### Procedure and materials

A detailed report of the procedure and the materials has been published elsewhere [11]. All subjects who provided written informed consent underwent a clinical examination carried out by experienced clinical psychologists. The clinical examination included both a standardized interview and self-report measures. The German version of the AUDIT [20] was used for the assessment of participants’ level of alcohol use. The presence of a substance use disorder diagnosis was assessed with section E of the German version of the *Structured Clinical Interview for DSM*-*IV Axis I Disorders* (SCID-I; [21]. The German version of the *Childhood Trauma Questionnaire* (CTQ; [19]) was administered to measure different types of childhood maltreatment occurring in the family context. The retrospective self-report questionnaire differentiates between emotional abuse, emotional neglect, physical abuse, physical neglect and sexual abuse. Parts of SCID-I were used to assess relevant psychiatric diagnoses. Besides section E (substance use disorders), the entire section A (mood disorders) and parts of section F (anxiety disorders: panic disorder, agoraphobia without history of panic disorder, agoraphobia with panic disorder, social phobia and generalized anxiety disorder) were used. DSM-IV diagnosis and severity of PTSD were assessed using the German version of the posttraumatic stress diagnostic scale (PDS; [22]).

The experimental task was identical for both samples and consisted of the priming task and the SAM-rating. AD subjects meeting inclusion criteria participated in the experimental task in an additional session. Control subjects participated in the experimental task immediately after the clinical examination. In the priming task, participants had to indicate after the presentation of a prime word whether the target was a real or a nonsense word by pressing the corresponding key. All real word stimuli consisted of German words from different categories: socially threatening words (SOC), physically threatening words (PHYS), alcohol related words (ALC) and neutral words (NEU). The priming task consisted of 16 practice trials and 192 experimental trials. Pairing of prime and target words resulted in eight stimulus conditions, which are outlined in Table 2. The priming task was immediately followed by the manipulation check. Participants were asked to rate all stimuli except the nonsense words regarding emotional valence and arousal using the self-assessment manikin self-report scale [23]. Further procedure details regarding the experimental design can be found in Potthast et al. [11].

**Table 2:** Stimulus conditions in the priming task

No.	Condition	Prime	Target
1	Test trials	SOC	ALC
2		PHYS	ALC
3	Baseline trials	SOC	NEU
4		PHYS	NEU
5		NEU	ALC
6	Task trials	SOC	NON
7		PHYS	NON
8		NEU	NON

*SOC* socially threatening words; *PHYS* physically threatening words; *ALC* alcohol related words; *NEU* neutral words; *NON* nonsense words

The study was approved by the Ethical Review Board of the University of Bielefeld. Control subjects were financially compensated for their participation.

### Data analysis

All statistical analyses are identical to those analyses used in our prior study [11]. Group comparisons in terms of demographic and psychometric properties were performed using χ^2^-tests and analyses of variance (ANOVA). The SAM ratings regarding valence and arousal were analysed with repeated-measures ANOVAs with word category (SOC, PHYS, ALC, NEU) as within-subject variable and group (AD + EA, AD + no EA, no AD) as between-subject variables. Bonferroni adjusted post hoc comparisons were used to compare word categories within each group. Differences in priming between the groups were tested using repeated measures analyses of covariance (ANCOVA) with age as covariate, separately calculated for SOC and PHYS. In both conditions, separate ANCOVAs were conducted comparing the test trials (SOC-ALC or PHYS-ALC) to the baseline trials with a neutral target (SOC-NEU or PHYS-NEU) and to the baseline trials with a neutral prime (NEU-ALC). With respect to our hypothesis of a specific priming effect, we expected a significant interaction between stimulus condition and group. In case of a significant interaction effect, the three groups were compared using an ANCOVA with planned comparisons (AD + EA vs. the other two subgroups). Difference scores (test trials minus baseline trials) were used as dependent variables, age was used as covariate.

## Results

### Self-assessment manikin

For the valence rating, the repeated measures ANOVA showed a significant main effect for word category, *F* (3, 51) = 45.71; *p* < 0.0001; *η* ^*2*^ = 0.73, a significant interaction of word category and group, *F* (6, 102) = 6.41; *p* < 0.0001; *η* ^*2*^ = 0.27, but no main effect of group, *F* (2, 53) = 2.38; *p* = 0.103; *η* ^*2*^ = 0.08. Regarding arousal, the ANOVA revealed a significant main effect for word category, *F* (3, 51) = 43.65; *p* < 0.0001; *η* ^*2*^ = 0.72, as well as a significant word category x group interaction, *F* (6, 102) = 6.84; *p* < 0.0001; *η* ^*2*^ = 0.29, but no main effect of group *F* (2, 53) = 2.81; *p* = 0.069; *η* ^*2*^ = 0.10. Bonferroni adjusted post hoc tests showed that within each group the socially threatening words as well as the physically threatening words were rated significantly more negative than neutral words (all *p* values <0.001). Additionally, within each group socially and physically threatening words were rated as more arousing than the neutral words. All *p* values were less than 0.01, except for the socially threatening words in the no AD group (*p* = 1.00). The manipulation check approved that socially and physically threatening cues elicited emotional reactions of the expected value.

### Priming task

**Fig. 1:**
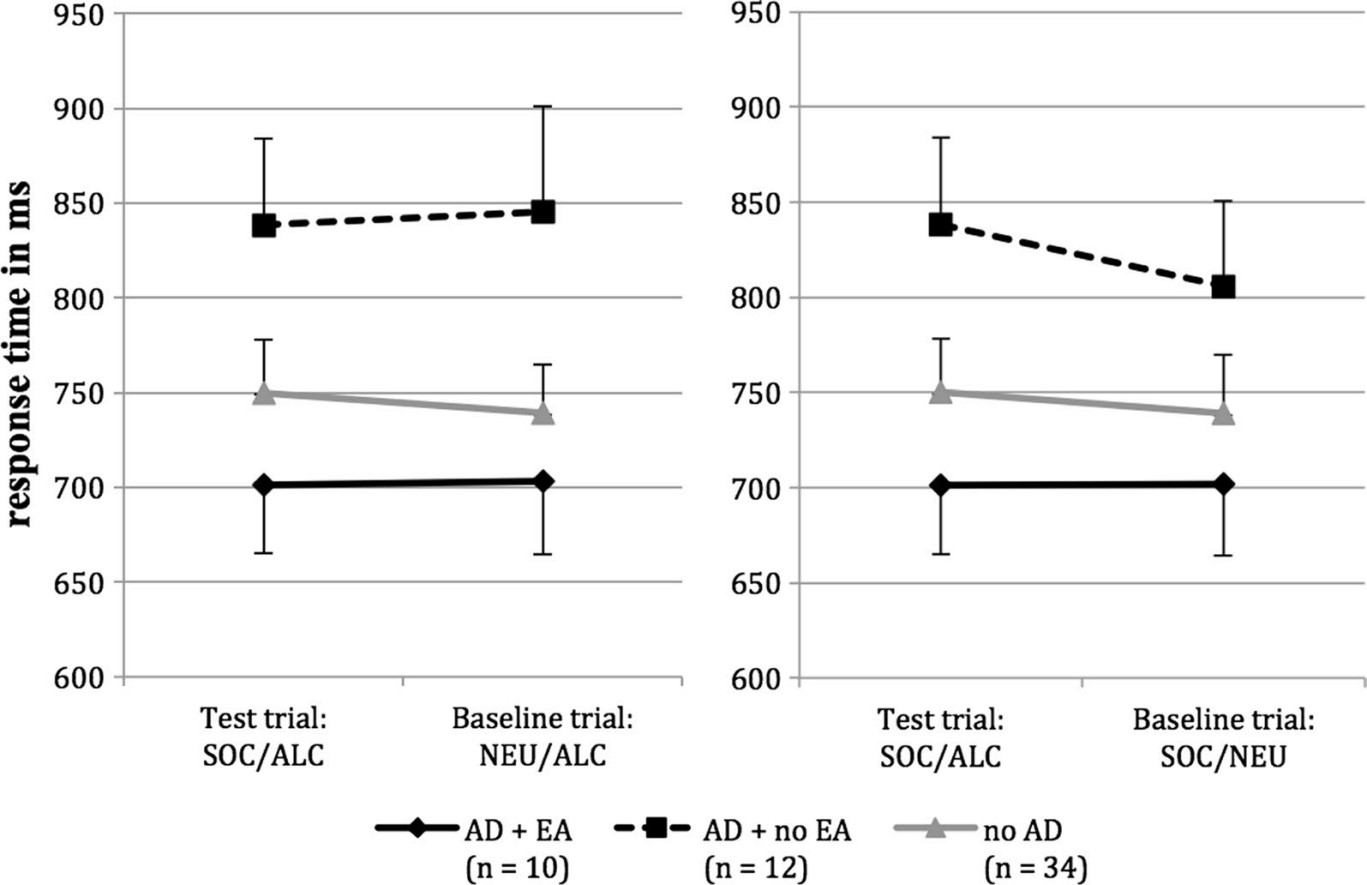
Average response time on socially threatening test trial (SOC-ALC) as contrasted to baseline trial with a neutral prime (NEU-ALC) on the left and to baseline trial with a neutral target (SOC-NEU) on the right. *AD* *+* *EA* subjects with alcohol dependence and emotional abuse experiences; *AD* *+* *no EA* subjects with alcohol dependence without emotional abuse experiences; *no AD* control subjects; *SOC* socially threatening words; *ALC* alcohol related words; *NEU* neutral words

The results of the ANCOVAS regarding the socially threatening words are illustrated in Fig. 1. Table 3 reports the corresponding mean response time and standard error in the test and baseline trials. As opposed to the hypothesis, the ANCOVA showed no significant effects when comparing the test trials to the neutral prime baseline [stimulus condition x group: *F* (2, 52) = 0.22; *p* = 0.807; *η* ^*2*^ = 0.01; stimulus condition: *F* (1, 52) = 0.03; *p* = 0.869; *η* ^*2*^ = 0.00; group: *F* (2, 52) = 2.47; *p* = 0.094; *η* ^*2*^ = 0.09]. Similarly, no significant effects were found when comparing the test trials to the neutral target baseline [stimulus condition x group: *F* (2, 52) = 0.57; *p* = 0.568; *η* ^*2*^ = 0.02; stimulus condition: *F* (1, 52) = 1.51; *p* = 0.224; *η* ^*2*^ = 0.03; group: *F* (2, 52) = 1.65; *p* = 0.202; *η* ^*2*^ = 0.06].

**Table 3:** Means and standard errors for response time in milliseconds on test and baseline trials

	AD + EA (n = 10)	AD + no EA (n = 12)	no AD (n = 34)
*Test trials*			
SOC/ALC	701.24 (36.28)	838.46 (45.36)	749.97 (28.21)
PHYS/ALC	719.77 (44.39)	865.97 (51.78)	744.80 (27.10)
*Baseline trials*			
NEU/ALC	703.34 (38.74)	845.49 (55.22)	739.24 (25.33)
SOC/NEU	701.25 (37.25)	805.86 (44.91)	738.97 (30.76)
PHYS/NEU	697.30 (41.01)	805.24 (48.24)	716.62 (24.26)

*AD* *+* *EA* subjects with alcohol dependence and emotional abuse experiences; *AD* *+* *no EA* subjects with alcohol dependence without emotional abuse experiences; *no AD* control subjects; *SOC* socially threatening words; *PHYS* physically threatening words; *ALC* alcohol related words; *NEU* neutral words

**Fig. 2:**
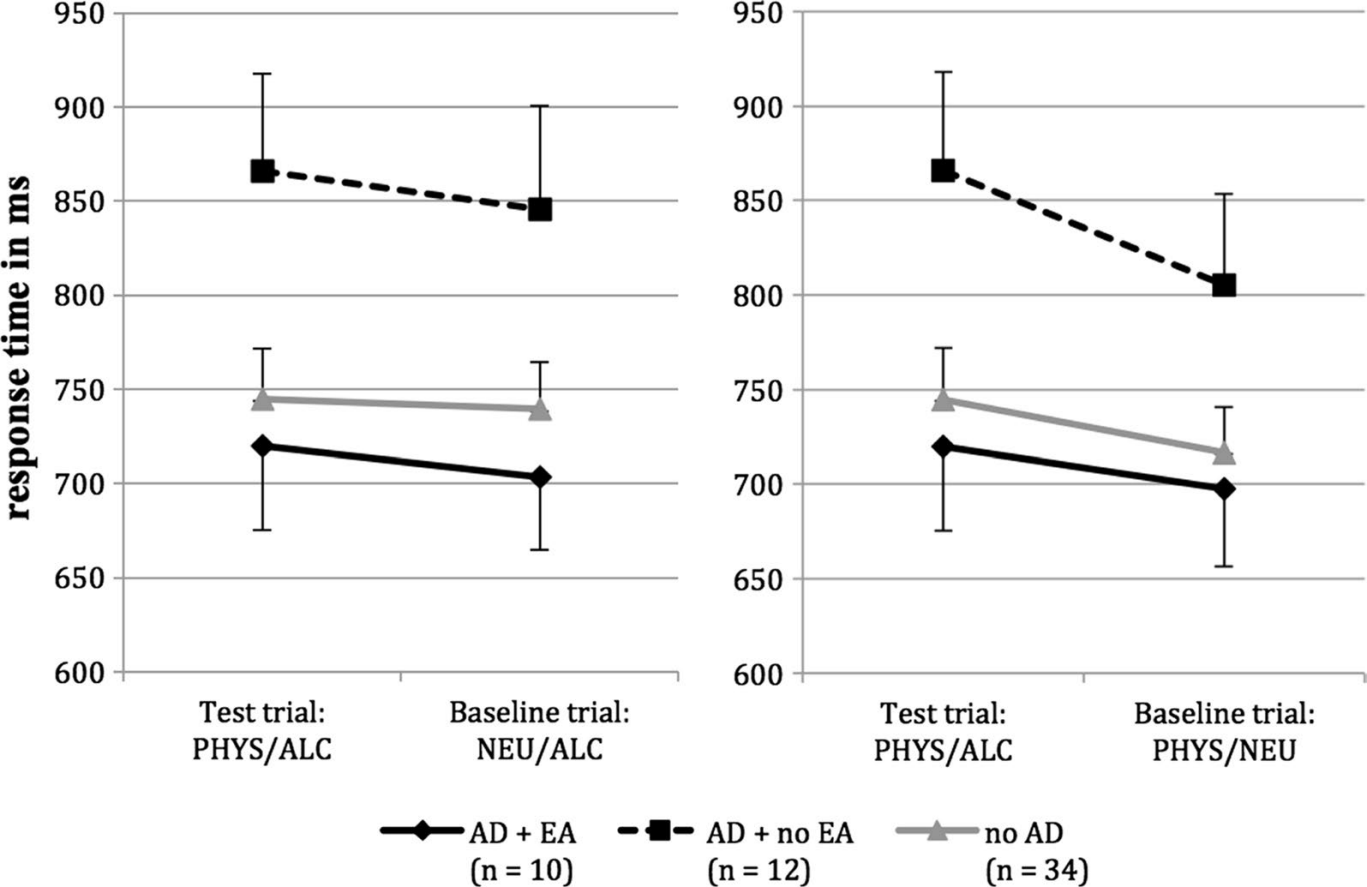
Average response time on physically threatening test trial (PHYS-ALC) as contrasted to baseline trial with a neutral prime (NEU-ALC) on the left and to baseline trial with a neutral target (PHYS-NEU) on the right. *AD* *+* *EA* subjects with alcohol dependence and emotional abuse experiences; *AD* *+* *no EA* subjects with alcohol dependence without emotional abuse experiences; *no AD* control subjects; *PHYS* physically threatening words; *ALC* alcohol related words; *NEU* neutral words

The results of the ANCOVAS concerning the physically threatening words are shown in Fig. 2 and the corresponding descriptive statistics in Table 3. Analogous to the socially threatening words, we found no significant effects when comparing the test trials to the neutral prime baseline (stimulus condition x group: *F* (2, 52) = 0.03; *p* = 0.975; *η* ^*2*^ = 0.00; stimulus condition: *F* (1, 52) = 0.547; *p* = 0.463; *η* ^*2*^ = 0.01; group: *F* (2, 52) = 2.59; *p* = 0.084; *η* ^*2*^ = 0.09). Similarly, the ANCOVA showed no significant effects when comparing the test trials to the neutral target baseline (stimulus condition x group: *F* (2, 52) = 0.64; *p* = 0.533; *η* ^*2*^ = 0.02; stimulus condition: *F* (1, 52) = 2.30; *p* = 0.135; *η* ^*2*^ = 0.04; group: *F* (2, 53) = 2.15; *p* = 0.127; *η* ^*2*^ = 0.08). As we did not find any significant interaction effects, no planned comparisons were conducted.

## Discussion

The purpose of the present study was to replicate our finding of a specific priming effect of maltreatment related words on alcohol words in a qualified detoxification sample. Our preceding study [11] indicated that only the AD + EA group showed a considerable reduction in response time to alcohol related target words after the presentation of maltreatment related prime words. The other two groups showed no reduction in response time. We concluded that the associative memory network can automatically be activated by child maltreatment related cues in subjects with AD and emotional abuse experiences. By means of the same priming paradigm we examined whether this automatic activation can also be found in subjects with AD and emotional abuse experiences being in qualified detoxification treatment instead of rehabilitation treatment. Interestingly, this pattern of results could not be replicated in qualified detoxification setting, as we did not find a specific priming effect in the present study.

This interesting finding of fundamentally different results within two distinct treatment settings deserves special emphasis. On the one hand, this discrepancy might be attributed to actually existing differences between both samples concerning the automatic activation of the associative network by child maltreatment related cues. This would be consistent to the possible selection effects regarding treatment settings, which are suggested by small transition rates from detoxification treatment to long-term rehabilitation treatment [13–16]. Weithmann and Hoffmann [17] indicated that patients proceeding to rehabilitation treatment seem to be more severely affected by their AD. They reported a more obsessive consumption pattern, more negative consequences of drinking and a more severe psychopathological burden as opposed to patients who quit after detoxification. Considering this finding and the fact that the specific priming effect was solely found within the rehabilitation setting, it might be speculated that the highly automatic activation of the associative network by child maltreatment related cues is closely related to AD severity. However, when compared to the rehabilitation sample in our prior study the present qualified detoxification sample does not show consistently higher levels of symptoms and impairments, in particular with respect to the AUDIT score as well as to comorbid psychopathology. Besides AD severity, the discrepancy could be caused by differences concerning the current treatment focus. Whereas detoxification treatment and especially qualified detoxification treatment predominantly aims at motivating patients to engage in a subsequent long-term rehabilitation treatment [12], patients being in rehabilitative treatment have to deal intensively with their drinking motives and triggers for craving. It is conceivable, that such therapeutic work might affect central elements of the associative memory network, thereby resulting in an initial reinforcement of the network associations. Additionally, the divergence of findings could be ascribed to alcohol related cognitive deficits, which probably differ in severity between qualified detoxification patients and those adhering to rehabilitation programs. It might be speculated that during the early qualified detoxification phase, cognitive functioning is more globally impaired thereby disguising a potential priming effect. Although cognitive deficits during both short- and long-term abstinence are well known [24], evidence regarding the rate of cognitive recovery is currently contradictory. Moreover, there are large interindividual differences in recovery process [25] impeding conclusions. Cognitive processes might also have been influenced by psychopharmacological medication for AD such as acamprosate and naltrexone, which was found in 30% of the qualified detoxification subjects but not within the rehabilitation sample of the prior study. As literature shows a significant reduction of cue-reactivity by acamprosate and naltrexone [26–28], a possible priming-effect might be disguised by such a medication. Due to the small sample size, a reanalysis of data after exclusion of qualified detoxifications subjects receiving AD medication was unfortunately not feasible.

Next to reasons related to treatment setting, the rather unexpected findings in the present study might also be attributed to methodological limitations. Firstly, the discrepancy might be assigned to the small sample size of the qualified detoxification subsamples (n = 10 and n = 12). Nevertheless, effect sizes for interactions between stimulus condition and group ranging from 0.00 to 0.02 are indicative that the absence of a priming effect does not stem from a lack of statistical power. Anyway, replication studies with larger qualified detoxification samples are warranted. Secondly, it has to be considered, that the qualified detoxification sample is drawn only from one institution, which is a day-unit institution in addition. Thus, a multicentric approach is preferable to evaluate the generalizability of findings for qualified detoxification services in general. Moreover, it remains to be clarified, how findings are generalizable to somatic detoxification treatments as the proportion of patients proceeding to qualified detoxification is limited. It would be particularly interesting to know, if findings are applicable to those patients, who quit after somatic detoxification. Albeit, the evaluation of a specific priming effect in a somatic detoxification sample would be hard to realize because of confounding factors. Finally, the unexpected findings in the present study might be attributed to a general vulnerability of priming effects regarding their replicability. In fact, a number of authors have found priming effects to be highly sensitive to variations in experimental features and subject populations [29, 30].

In summary we can say that additional work is required to determine the generalizability of the specific priming effect of maltreatment related words in patients with AD as suggested by our previous study [11]. Replication studies in different treatment settings, with multicentric samples and with large sample sizes are needed to clarify reasons for the discrepant results in the present study, for instance by considering potential confounding factors, such as AD severity or level of alcohol-related or medication-related cognitive deficits.

At the current stage, we cannot draw valid conclusions concerning automatic activation of the associative memory network as a potential underlying mechanism of the link between child maltreatment and AD. The present results show that prior evidence arguing for the existence of such a mechanism might be associated to the specific treatment setting of the study sample. Further research is needed to evaluate the robustness and generalizability of related findings across treatment settings, treatment institutions and populations thereby allowing important implications regarding the treatment of AD.

## Abbreviations

AD: alcohol dependence
EA: emotional abuse
